# Diagnostic value of transcranial doppler to predict delayed cerebral ischemia after aneurysmal subarachnoid hemorrhage: To predict delayed cerebral ischemia

**DOI:** 10.1007/s00701-024-06164-1

**Published:** 2024-06-29

**Authors:** J. Joep van der Harst, Jan Willem J. Elting, Johanna Hijlkema, Nic J. G. M. Veeger, Carlina E. van Donkelaar, J. Marc C. van Dijk, Maarten Uyttenboogaart

**Affiliations:** 1 Departments of Neurology, University of Groningen, University Medical Center Groningen, Groningen, The Netherlands; 2 Department of Epidemiology, Uversity of Groningen, University Medical Center Groningen, Groningen, The Netherlands; 3 Department of Neurosurger, Uversity of Groningen, University Medical Center Groningen, Groningen, The Netherlands

**Keywords:** Subarachnoid Hemorrhage, Transcranial Doppler, Delayed Cerebral Ischemia, Modified Rankin scale, SAFIRE

## Abstract

**Background:**

Transcranial Doppler (TCD) is a technique to assess blood flow velocity in the cerebral arteries. TCD is frequently used to monitor aneurysmal subarachnoid hemorrhage (aSAH) patients. This study compares TCD-criteria for vasospasm and its association with Delayed Cerebral Ischemia (DCI). An overall score based on flow velocities of various intracranial arteries was developed and evaluated.

**Methods:**

A retrospective diagnostic accuracy study was conducted between 1998 and 2017 with 621 patients included. Mean flow velocity (MFV) of the cerebral artery was measured between 2–5 days and between 6–9 days after ictus. Cutoff values from the literature, new cutoff values, and a new composite score (Combined Severity Score) were used to predict DCI. Sensitivity, specificity, and area under the curve (AUC) were determined, and logistic regression analysis was performed.

**Results:**

The Combined Severity Score showed an AUC 0.64 (95%CI 0.56-.71) at days 2–5, with sensitivity 0.53 and specificity 0.74. The Combined Severity Score had an adjusted Odds Ratio of 3.41 (95CI 1.86–6.32) for DCI. MCA-measurements yielded the highest AUC to detect DCI at day 2–5: AUC 0.65 (95%CI 0.58–0.73). Optimal cutoff MFV of 83 cm/s for MCA resulted in sensitivity 0.73 and specificity 0.50 at days 2–5.

**Conclusion:**

TCD-monitoring of aSAH patients may be a valuable strategy for DCI risk stratification. Lower cutoff values can be used in the early phase after the ictus (day 2–5) than are commonly used now. The Combined Severity Score incorporating all major cerebral arteries may provide a meaningful contribution to interpreting TCD measurements.

**Supplementary Information:**

The online version contains supplementary material available at 10.1007/s00701-024-06164-1.

## Introduction

Recently, the *2023 Guideline for the Management of Patients with Aneurysmal Subarachnoid Hemorrhage* was issued by the American Heart Association & American Stroke Association. The guideline clearly states that in addition to clinical monitoring by trained nurses, there is a place for diagnostic modalities as transcranial Doppler (TCD), Computed Tomography Angiography (CTA), and Computed Tomography Perfusion (CTP). These diagnostic modalities may provide timely information about the risk of developing delayed cerebral ischemia (DCI) [12].

Proven treatment for the prevention of DCI is nimodipine, with a Number Needed to Treat (NNT) of 8 and a NNT of 13 to prevent poor neurological outcomes [9]. Admission to intensive care or neurovascular unit with neurologically trained nurses is advised. Declined level of consciousness or emerging focal deficits are indications for intensive treatment to prevent DCI [12, 28]. Treatment options in case of symptomatic vasospasm are raising the arterial blood pressure while maintaining euvolemia. In severe cases, intra-arterial vasodilator therapy or cerebral angioplasty may also be reasonable to reduce the risk of DCI [12, 17] .

Monitoring with TCD could have important therapeutic consequences. It has the advantage of bedside application, relatively low costs, and no radiation exposure. As such, TCD can be frequently performed during extended time intervals [15]. TCD measures the flow velocities of intracranial blood vessels, which are inversely related to the diameter of a blood vessel (Bernoulli’s principle). Consequently, TCD-measured flow velocities are correlated with vasospasm and can thus be used as a surrogate measure [18]. Limitations in this assumption are the length of a vessel, blood viscosity, and friction pressure loss, which may also affect flow velocity according to Poiseuille’s law. In addition, cerebral blood flow (CBF) may change due to systemic hemodynamics and impaired autoregulation. The proportional relationship of flow velocity is therefore not conclusive [1, 2]. In practice, with a low-grade stenosis of less than 50%, there is no increase in flow velocity, and flow velocity decreases with near occlusion, as Spencer’s curve shows [3]. Also, vasospasm is not directly correlated to the occurrence of DCI. It is known that patients may develop DCI in the absence of prior vasospasm [6, 32]. This aligns with the complex pathophysiology of DCI, involving many factors apart from vasospasm [8] .

The current study aims to compare TCD-criteria for vasospasm [13, 16, 19, 27, 33] and its association with DCI. Also, the most optimal cutoff values of flow velocities associated with DCI are assessed. Finally, an overall score based on flow velocities of various intracranial arteries (Combined Severity Score) is developed and evaluated.

## Materials and methods

### Patient selection

This study retrospectively analyzed data from a prospectively kept observational cohort admitted to our academic neurovascular unit (tertiary care) from November 1998 to December 2017. Consecutive adult aSAH-patients with at least two TCD measurements between days 2–5 and 6–9 were included. In addition, availability of follow-up data on functional outcomes and DCI was an inclusion criterion. The research ethical board approved the study, waiving informed consent. The *no-objection* register was consulted for each patient.

### Patient assessment

Baseline characteristics were gender, age, hypertension, World Federation of Neurosurgical Societies grading after resuscitation (rWFNS), modified Fisher scale, aneurysm location, size aneurysm (mm), treatment type, cerebrospinal fluid (CSF) drainage, DCI, and modified Rankin Scale (mRS). SAFIRE was assessed as a representation of the patient characteristics, as a higher SAFIRE grade is a predictor of a higher likelihood of an unfavorable outcome. The SAFIRE grading scale consists of 4 items: aneurysm size, age, modified Fisher scale, and rWFNS [31]. The rWFNS was determined by the best neurological WFNS score within 12 h after neurological resuscitation [31]. Modified Fisher scale was based on CT-head on admission [11]. Location of an aneurysm was dichotomized to anterior and posterior circulation (Supplement Table S1). Treatment type could be coiling, clipping, or none. CSF fluid drainage consisted of an external ventricular drain, external lumbar drain, cisternal drain, and ventriculoperitoneal drain. Functional outcome (mRS) was obtained at two months follow-up or at discharge if follow-up was unavailable. Functional outcome was dichotomized in mRS ≤ 3 (favorable) and mRS > 3 (unfavorable) [23]. All patients were treated according to national SAH-management guidelines, based on American Heart Association guidelines and European Stroke Organization Guidelines. The protocol included oral nimodipine, fluid management to prevent hypovolemia, and frequent evaluation of the neurologic condition [12, 28] .

DCI was used as an outcome variable, defined by a reduced level of consciousness and/or new focal neurological deficits and/or new infarction on follow-up imaging (CT or MRI) after exclusion of other causes such as epilepsy, hydrocephalus, and systemic factors [32]. If DCI was suspected, blood pressure augmentation was induced in the intensive care unit (ICU) [30] .

### TCD Examinations and variables

TCD was performed daily or every other day by experienced Doppler technicians. Recordings of the anterior and posterior circulations were obtained through a transtemporal windows using a 2-MHz handheld transducer probe (Pioneer TC 4040; Nicolet Biomedical, Inc., Madison, WI). The middle cerebral artery (MCA) insonation depth varied between 50–60 mm, for the anterior cerebral artery (ACA) between 65–70 mm, and the posterior cerebral artery (PCA) 55–70 mm. Arterial mean flow velocity (MFV) was recorded. Two time intervals were used for analysis, day 2–5 and day 6–9. The highest values of separate arteries (left/right, proximal/distal) at the measurement of maximum MFV of the MCA within these time frames were used for further analysis. The Lindegaard ratio was calculated as the MCA-MFV divided by the ipsilateral extracranial MFV of the internal carotid artery (ICA) [16]. The Sloan ratio was calculated as ACA-MFV divided by ipsilateral ICA-MFV [13]. Common TCD criteria for vasospasm were used: MCA-MFV > 120 cm/s; Lindegaard ratio > 3; ACA-MFV > 120 cm/s; Sloan ratio > 4; and PCA-MFV > 110 cm/s [13, 19, 33] .

We developed the Combined Severity Score to obtain a collective representation of the accomplished TCD measurements. Common definitions from the literature on the severity of vasospasm at the MCA, ACA, and PCA were applied. This score is largely based on the vasospasm severity classification used by Snider et al. 2022 [27]. The Combined Severity Score was calculated by classifying and subsequently summating vasospasm of the MCA, ACA, and PCA into none, mild, moderate, and severe, respectively, using 0–3 points for each artery (Table 1). When MCA, ACA, and PCA scores are summated the Combined Severity Score can have a maximum of 9 points (Table 1.).

**Table 1:** Transcranial Doppler Vasospasm Severity Score

			Anterior Cerebral Artery		Posterior Cerebral Artery	
MFV (cm/s)	LR	points	MFV(cm/s)	points	MFV(cm/s)	points
120–149	> 3	1 (mild)	100–129	1 (mild)	80–119	1 (mild)
150–199	> 3	2 (moderate)	130–149	2 (moderate)	120–159	2 (moderate)
> 200	3–6	2	> 150	3 (severe)	> 160	3 (severe)
> 200	> 6	3 (severe)				

TCD, indicates Transcranial Doppler; MFV, Mean Flow Velocity; LR, Lindegaart ratio

### Statistical analysis

All statistical analyses were performed in R version 4.2.2 under RStudio 2023 version 03.1 + 446 (R Foundation for Statistical Computing, Vienna, Austria; http://www.r-project.org).

Univariate analysis was performed for DCI. Given the non-gaussian distribution, the Mann–Whitney U-test was used for medians of groups. For categorical data, contingency tables, chi-squared tests, or Fisher exact tests were used. The diagnostic value of predicting DCI by the different TCD parameters is determined by Receiver Operator Characteristic (ROC) analyses with the area under the curve (AUC) and 95% confidence intervals (CI) to quantify diagnostic accuracy. AUCs of correlated ROC curves were compared using DeLong’s test [7]. Optimal TCD cutoff values were established with the Youden index. In addition, usual TCD cutoff values were used to predict DCI. With these cutoff values, diagnostic accuracy was assessed with contingency analysis, including sensitivity, specificity, and accuracy ((true positive + true negative)/total number of patients). Statistical significance was determined by a two-sided probability value of < 0.05.

Univariate analyses were used to select independent predictors of DCI with a p < 0.2 for entering multivariable logistic regression analysis. Odds ratio (OR) and 95%CI were presented. From TCD parameters, only the best performing and of practical use in the clinical practice were included in the multivariable logistic regression analysis.

## Results

### Patient assessment

In the neurovascular database, 1247 aSAH patients had at least two TCD measurements available. For days 2–5, 885 complete measurements, and for days 6–9, 1021 complete measurements were present. Combining the two-time intervals and including follow-up data on functional outcome, 621 patient records were complete (see supplement Flow Diagram S3), of which 109 (18%) had an unfavorable functional outcome (mRS > 3). Fifty-five (9%) had developed DCI. All baseline characteristics are presented in Table 2.

**Table 2:** Baseline characteristics of the study Population

	Overall N = 621
Female	428 (69%)
Age, years, median (IQR)	54 (45, 62)
Hypertension	147 (24%)
World Federation of Neurosurgical Societies grading after neurological resuscitation	
I	273 (44%)
II	183 (29%)
III	24 (3.9%)
IV	104 (17%)
V	37 (6.0%)
Modified Fisher scale*	
0	8 (1.4%)
1	138 (23%)
2	133 (23%)
3	103 (17%)
4	207 (35%)
Location aneurysm*	
Anterior	416 (67%)
Posterior	200 (32%)
Size aneurysm, mm, median (IQR)	6.0 (4.0, 9.0)
Treatment	
Coiling	343 (58%)
Clipping	247 (42%)
Cerebrospinal fluid drainage	
No drain	188 (34%)
Drain before treatment	252 (41%)
Drain after treatment	181 (29%)
SAFIRE grade	
1	229 (37%)
2	174 (28%)
3	113 (18%)
4	98 (16%)
5	7 (1.1%)
Delayed Cerebral Ischemia	
DCI	55 (8.9%)
No DCI	566 (91%)
Functional Outcome (Binomial modified Rankin Scale)	
mRS > 3	109 (18%)
mRS ≤ 3	512 (82%)

IQR indicates interquartile range; SAFIRE, size aneurysm, age, Fisher grade, world federation of neurosurgical societies grading after resuscitation; mRS, modified Rankin Scale; DCI, Delayed Cerebral Ischemia

* For Modified Fisher scale, there were 32 missing values, for location aneurysm 5, for treatment 31

Of the 626 excluded patients, 192 (31%) had an unfavorable functional outcome, and 68 (11%) had DCI (see supplement Table S3).

### TCD Examinations and variables

**Fig. 1:**
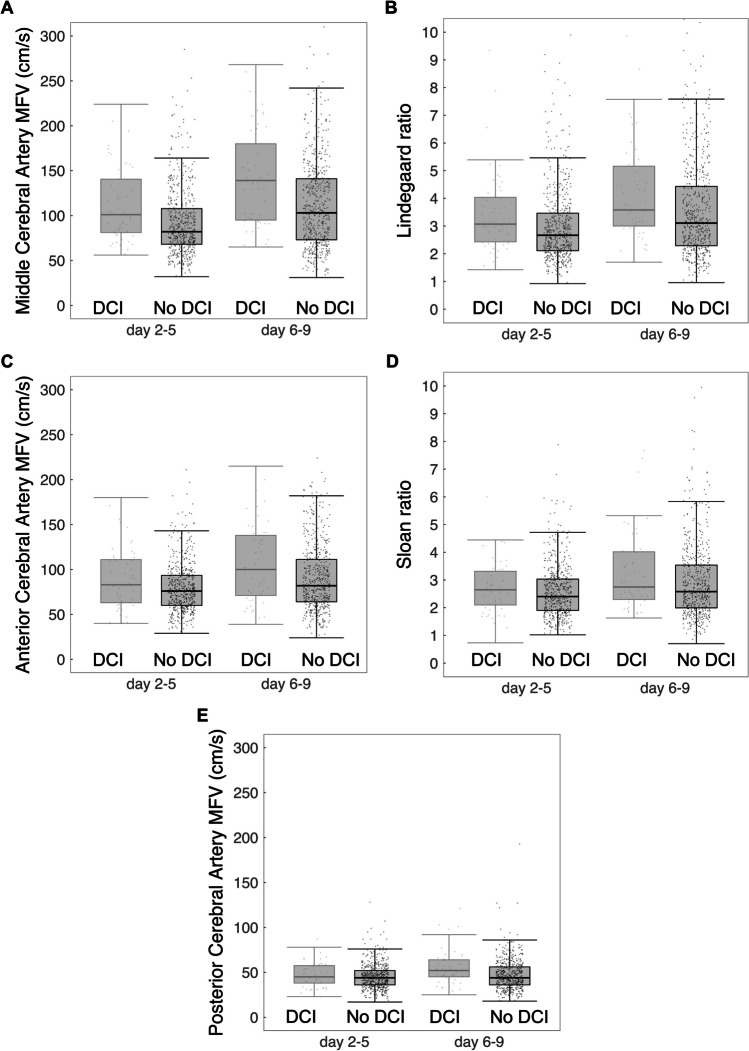
Box plots days 2–5 and days 6–9 for Delayed Cerebral Ischaemia (DCI) and No DCI. A, Middle Cerebral Artery. B, Lindegaard ratio. C, Anterior Cerebral Artery. D, Sloan ratio. E, Posterior Cerebral Artery

**Fig. 2:**
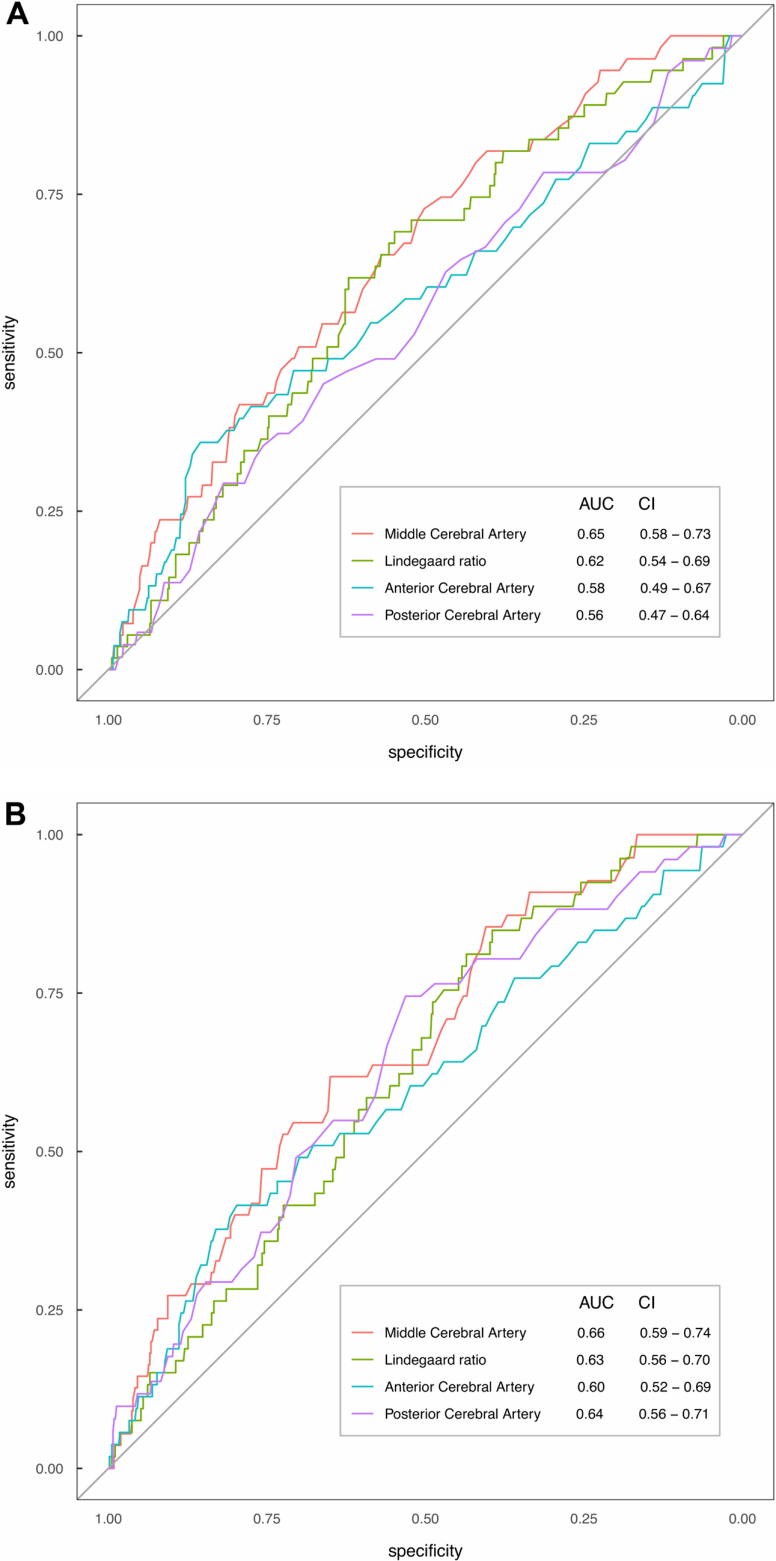
ROC analysis (sensitivity vs specificity) for Delayed Cerebral Ischaemia (DCI). A, days 2–5. B, days 6–9. ROC indicates receiver operating characteristic; AUC, Area Under Curve; CI, Confidence Interval

Median MFV in MCA and ACA were significantly higher in the DCI group in both time frames (days 2–5 and 6–9). Median MFV in PCA was significantly higher in the DCI group for days 6–9 (Fig. 1; Supplemental Table S5). Highest AUCs were found in the MCA: on days 2–5, AUC 0.65 (95%CI 0.58–0.73), and on days 6–9, AUC 0.66 (95%CI 0.69–0.74) [Fig. 2]. The optimal cutoff for the MCA on days 2–5 was 83 cm/s, and on days 6–9, 129 cm/s. This resulted in a sensitivity of 0.73 and 0.62, respectively, and a specificity of 0.50 and 0.65, respectively. Other MFVs and indexes were comparable or less (Table 3).

**Table 3:** TCD mean flow velocity and ratios predicting delayed cerebral ischemia

	AUC	CI	optimal cutpoint	sensitivity	specificity	accuracy
Middle Cerebral Artery Mean Flow Velocity (cm/s)						
Day 2–5	0.65	0.58—0.73	83	0.73	0.50	0.52
Day 6–9	0.66	0.59 – 0.74	129	0.62	0.65	0.65
Lindegaard Ratio						
Day 2–5	0.62	0.54—0.69	2.8	0.69	0.55	0.56
Day 6–9	0.63	0.56—0.70	2.9	0.81	0.44	0.47
Anterior Cerebral Artery Mean Flow Velocity (cm/s)						
Day 2–5	0.58	0.49—0.67	106	0.36	0.85	0.81
Day 6–9	0.60	0.52—0.69	119	0.42	0.80	0.76
Sloan Ratio						
Day 2–5	0.55	0.47—0.64	2.5	0.58	0.54	0.54
Day 6–9	0.58	0.50—0.66	1.8	0.96	0.18	0.25
Posterior Cerebral Artery Mean Flow Velocity (cm/s)						
Day 2–5	0.56	0.47—0.64	57	0.29	0.82	0.77
Day 6–9	0.64	0.56—0.71	46	0.75	0.53	0.55

TCD indicates Transcranial Doppler; AUC, Area Under Curve; CI, Confidence Interval

Out of the common TCD criteria, MCA-MFV > 120 cm/s with a Lindegaard ratio > 3 at day 2–5 had the highest AUC (0.58; 95%CI 0.52–0.64), resulting in sensitivity 0.33 and specificity 0.83. For the ACA, ACA-MFV > 120 cm/s with a Sloan ratio > 4 at days 6–9 had an AUC of 0.57 (95%CI 0.50–0.65), resulting in a sensitivity 0.29 and specificity 0.86. The Combined Severity Score had the highest AUC of all criteria at day 2–5 (0.64; 95%CI 0.56-0.71). With an optimal cutoff of ≥ 1 at days 2–5, the Combined Severity Score had a sensitivity 0.53 and specificity 0.74. This was slightly lower at days 6–9 (Table 4; Supplemental Table S6 and S7). The AUC of the Combined Severity Score on day 2–5 0.64 (95%CI 0.56-0.71) did not significantly differ with the AUC of the MCA 0.65 (95%CI 0.58–0.73) on day 2–5 (p = 0.47).

**Table 4:** TCD definitions predicting delayed cerebral ischemia

	AUC	CI	sensitivity	specificity	accuracy
Middle Cerebral Artery Mean Flow Velocity > 120 cm/s & Lindegaard Ratio > 3					
Day 2–5	0.58	0.52—0.64	0.33	0.83	0.79
Day 6–9	0.56	0.49—0.63	0.47	0.65	0.63
Anterior Cerebral Artery Mean Flow Velocity > 120 cm/s & Sloan ratio > 4					
Day 2–5	0.52	0.47—0.56	0.08	0.95	0.87
Day 6–9	0.57	0.50—0.65	0.29	0.86	0.82
Posterior Cerebral Artery Mean Flow Velocity > 110 cm/s					
Day 2–5	0.50	0.50—0.50	0.00	1.00	0.91
Day 6–9	0.51	0.49—0.53	0.02	0.99	0.91
Combined Severity Score					
Day 2–5 Optimal Cutpoint ≥ 1	0.64	0.56—0.71	0.53	0.74	0.72
Day 6–9 Optimal Cutpoint ≥ 3	0.61	0.53—0.69	0.35	0.82	0.78

TCD, indicates Transcranial Doppler; AUC, Area Under Curve; CI, Confidence Interval

### Univariate and multivariable analysis

Univariate statistical analysis for DCI versus no DCI showed a significant difference for rWFNS (p = 0.027), CSF drainage (p = 0.043), SAFIRE grade (p = 0.049), and functional outcome (p < 0.001). Patients with rWFNS grade IV and V had higher percentages of DCI, 20% and 16%, vs. patients without DCI, 16% and 4.9% (p = 0.027). CSF drainage after treatment was more frequent in patients with DCI (p = 0.043). Patients with DCI had higher SAFIRE grades (p = 0.049) and 49% unfavorable outcome, versus 14% in patients without DCI (p < 0.001; supplement Table S4).

The following variables were selected for the multivariable logistic regression analysis: rWFNS (p = 0.045), modified Fisher scale (p = 0.079), CSF drainage (p = 0.051), and Combined Severity Score on day 2–5 (p =  < 0.001). The Combined Severity Score of days 2–5 predicted DCI with an adjusted Odds Ratio (aOR) of 3.41 (95% CI 1.86–6.32; p =  < 0.001), while after adjustment, rWFNS, modified Fischer scale, and CSF drainage were no longer significantly associated with DCI, (Table 5).

**Table 5:** Logistic regression analysis for DCI after aneurysmal SAH

n = 621	Univariate Analysis			Multivariable Analysis *		
	OR	95% CI	PValue	aOR	95% CI	PValue
Female vs Male	0.77	0.44, 1.39	0.38			
Age (year)			0.78			
> 65	reference					
50–65	0.80	0.38, 1.78	0.57			
< 50	0.97	0.46, 2.15	0.94			
Hypertension	1.11	0.57, 2.05	0.74			
rWFNS			0.045			0.31
I	reference					
II	0.97	0.46, 1.98	0.93	0.55	0.23, 1.26	0.16
III	1.15	0.18, 4.31	0.86	0.91	0.13, 3.65	0.90
IV	1.50	0.67, 3.19	0.31	0.81	0.32, 1.98	0.65
V	4.07	1.63, 9.60	0.002	1.64	0.57, 4.46	0.34
modified Fisher scale			0.079			0.34
1	reference					
2	1.44	0.52, 4.14	0.48	1.50	0.51, 4.52	0.46
3	2.14	0.79, 6.07	0.14	1.76	0.61, 5.25	0.30
4	2.73	1.21, 7.0	0.023	2.42	0.91, 7.04	0.086
Location aneurysm						
Anterior vs Posterior	0.92	0.50, 1.66	0.80			
Aneurysm diameter			0.62			
< 10 mm	reference					
10–19.9 mm	0.98	0.44, 1.99	0.96			
≥ 20 mm	2.33	0.35, 9.39	0.29			
Coiling vs. Clipping	1.26	0.70, 2.24	0.43			
Drain			0.051			0.24
No drain	reference					
Before treatment	1.20	0.57, 2.60	0.64	0.83	0.36, 1.91	0.65
After treatment	2.24	1.11, 4.78	0.029	1.53	0.66, 3.65	0.33
Combined Severity Score (day 2–5)						
≥ 1	3.15	1.80, 5.55	< 0.001	3.41	1.86, 6.32	< 0.001
Combined Severity Score (day 6–9)						
≥ 3	2.43	1.32, 4.37	0.004			

OR indicates Odds Ratio; aOR, adjusted Odds Ratios; CI, Confidence Interval; rWFNS, World Federation of Neurosurgical Societies grading after neurological resuscitation

*Effect estimates from the multivariable regression are adjusted for all other variables in the model

## Discussion

The Combined Severity Score based on common vasospasm criteria has a higher AUC, sensitivity, and specificity than the common vasospasm criteria of individual arterial segments. Common TCD-criteria for vasospasm in individual arteries did not reliably predict DCI. In early measurements (days 2–5), sensitivity improved significantly with lower MFV-cutoffs determined as the new optimal cutoff values in the ROC curves. In general, flow velocity increases over time, but specific treatments may also influence flow velocities [4]. Nevertheless, the lower flow velocities in first measurements had only slightly better predictive value for the development of DCI. This could be explained by the fact that early (prodromal) changes may be more pathological than late. Late increases in flow velocities maybe also physiological and even harbour protective effects. In addition, treatment will influence the development of DCI. It is known that vasospasm or high flow velocities do not necessarily lead to DCI and that DCI can also occur without vasospasm [6, 24, 25].The clinical characteristics rWFNS, SAFIRE grade, and ventricular drainage were associated with occurrence of DCI. The presence of a CSF drain after treatment was more common in patients with DCI, but this was probably explained by the fact that these patients were in a worse clinical condition. As previous studies have shown, DCI is associated with poor functional outcomes, which is also demonstrated in the current study [8, 31] .

Our study shows that a high SAFIRE score is associated with DCI. This was as expected since the SAFIRE score was developed to predict the risk for unfavorable functional outcomes [8]. Since the SAFIRE score is a composite score designed to predict functional outcomes, it was not included in the multivariable analysis [31]. Instead, components of the SAFIRE score were used for multivariable analysis.

The percentage favorable outcome in our study was relatively high among the included patients, suggesting that patients with poor outcomes had less often TCD measurements. However, DCI was not less common in the included patients 55 (9%) compared to the excluded patients 68 (11%) Since the study's primary outcome measure was the prediction of DCI, we consider that the somewhat overrepresented good favorable outcome did not affect the study results.

### Comparison

One of the notable findings in our study is that in the early timeframe (days 2–5), MCA-MFV had the highest AUC with a much lower optimal cutoff value (83 cm/s) than the conventional 120 cm/s [13]. This aligns with the concept that the MFV is usually lower directly after the ictus and increases in the following days [5, 20, 27]. The AUC of the Combined Severity Score was higher than for the common criteria, with improved sensitivity, particularly on days 2–5, contributing to a more accurate prediction of the risk for DCI.

In previous studies, a CTA vasospasm sum score of several cerebral arteries predicted DCI and functional outcomes better than vasospasm in any individual artery [29, 30]. A recent study by Snider et al. supports the importance of considering the severity of vasospasm on TCD for predicting DCI. In this study, an increase in vasospasm severity was associated with a hazard ratio of 1.7 (95% CI 1.1–2.4) for DCI [27]. This is consistent with the findings in our study in which TCD vasospasm severity criteria can be valuable in predicting the likelihood of DCI. However, instead of using the most affected artery for further analysis, the severity of a variety of arteries was weighted for analysis in our study.

A meta-analysis reported a sensitivity of 90% (95% CI 77%–96%) and specificity of 71% (95% CI 51%–84%) for TCD in predicting DCI [14]. However, it is essential to consider the individual studies included in this meta-analysis. One heavy-weighing study in this meta-analysis (n = 786 of the total n = 2870; 27%) aimed not to predict DCI but focused on the agreement between TCD and angiography as the gold standard. When looking at the 6% infarction on CT in this study, sensitivity and specificity for DCI would probably be much lower than used in the meta-analysis [10, 14]. Our study aligns with the findings of another study with 441 patients. In this study, a sensitivity 0.63 and specificity 0.52 was reported with an adjusted odds ratio of 1.74 (95% CI 1.06–2.86) for MFV > 120 cm/s in predicting DCI [6] .

### Strengths and limitations

A major strength of our study is the relatively large sample size (n = 621), in which multiple TCD examinations were conducted. There were enough patients with DCI to perform reliable (multivariable) statistical analyses. We used a practical approach with the Combined Severity Score to incorporate TCD measurements of multiple arteries to predict DCI. Also, the timing of the measurements in two frames (days 2–5 and 6–9) is useful for clinical practice since these time intervals would have the most clinical consequences. Moreover, we demonstrated that the cutoff scores to predict DCI should be different at days 2–5 versus days 6–9. As such, a next step would involve including time-dependent cutoffs and probably patient-specific (age, sex) for revising the Combined Severity Score.

In this study, the AUC of the MCA-MFV and the Combined Severity Score were moderate and did not differ significantly. The MCA determines the Combined Severity Score to a large extent in our data. Advantage of the Combined Severity Score is that the MCA-MFV is weighted, and other arteries were included. Other arteries are relevant to include in the model, as vasospasm and DCI can occur throughout the brain, but this is less evident in the current study data. DCI has a complex pathophysiology of vascular dysfunction, inflammation, and spreading depolarization. TCD provides insight into some of the vascular dysfunction, particularly macrovascular dysfunction of proximal arteries [8]. Microvascular dysfunction, microthrombosis, and autoregulatory failure remain out of the scope of stand-alone TCD measurements. However, the Combined Severity Score could contribute to a risk prediction model within a framework. In such multimodal risk model, patient characteristics, clinical variables, and other modalities, such as CTA and NIRS, can be of added value. We recommend that future (intervention) studies continue to perform TCD measurements and incorporate the Combined Severity Score. First, it can be used in a multimodal model to select patients for vasospasm treatment. Second, it can be used as a secondary outcome measure. It can be useful as a secondary outcome measure because the Combined Severity Score represents the vascular status of major cerebral arteries, provides pathophysiological insight into the effects of treatment strategies, and is related to DCI, which is related to functional outcomes.TCD has specific limitations. First, TCD measurements were measured as snapshots in our study. In snapshots, blood flow velocities are measured regularly for a few minutes per artery. Since hemodynamics can fluctuate, high flow velocities may have been missed. Fluctuations also provide information about impaired autoregulation, which is a risk factor for DCI as well. Continuous TCD measurements would likely offer valuable information [8, 22]. Second, a more general limitation of TCD is that approximately 10–20% of patients may have an insufficient temporal bone window. Alternative forms of monitoring may be considered for these patients [21]. In addition, TCD measurements depend on the expertise and experience of the sonographer, which is important for inter and intra-observer agreement. With proper expertise and regular exposure, an intra-class correlation coefficient of 0.9 (95% CI 0.81–0.99) can be achieved [26] .

## Conclusion

Our study demonstrates that regular TCD monitoring of aSAH patients is valuable for DCI risk stratification. Increased MFV was significantly more common in patients who developed DCI, with lower cutoff values in the early phase after the ictus (days 2–5). The Combined Severity Score incorporates all major cerebral arteries and may provide a meaningful contribution to the interpretation of TCD measurements.

## Comments:

The authors present a novel vasospasm severity score for TCD as a tool to predict DCI after aSAH. TCD is well established in diagnostics of large vessel vasospasm, however, evaluation criteria vary between departments and some practice merely insonation of the MCAs and draw treatment conclusions from that. Single vessel approaches may be misleading and may in part be the cause to why the value of TCD has been doubted. In this respect the approach with a severity score considering the major cerebral arteries and the Lindegaard ratio is to be applauded. Prospective implementation of this severity score into daily routine would establish its value as an outcome measure and tool in selecting patients for invasive vasospasm treatment.

Angelika Sorteberg

Oslo, Norway

## Supplementary Information

Below is the link to the electronic supplementary material.

Supplementary file1 (PDF 313 KB)

## Data Availability

The data that support the findings of this study are available from the corresponding author upon reasonable request.

## Code availability

Code will be made available on request by contacting the corresponding author.
