# Supplementary material for: Diagnostic value of transcranial doppler to predict delayed cerebral ischemia after aneurysmal subarachnoid hemorrhage: To predict delayed cerebral ischemia
Source: Acta Neurochir (Wien). 2024 Jun 29;166(1):278. doi: 10.1007/s00701-024-06164-1 (PMC11217085; doi:10.1007/s00701-024-06164-1)
Supplement: Supplementary file 1 — Supplementary file1 (PDF 313 KB) [file 701_2024_6164_MOESM1_ESM.pdf]

SUPPLEMENTAL MATERIAL

Table of Contents

SUPPLEMENTAL MATERIAL **1**

Table S1. Dichotomized location of aneurysm in anterior and posterior circulation. *1*

Figure S2. Flow diagram of included patients. *2*

Table S3. Functional Outcome and Delayed Cerebral Ischemia for included and excluded patients. *2*

Table S4. Patient Characteristics for DCI and functional outcome. *3*

Table S5. TCD Variables for Delayed Cerebral Ischemia and no Delayed Cerebral Ischemia. *4*

Table S6. Combined Severity Score Day 2-5. .... *4*

Table S7. Combined Severity Score Day 6-9. .... *4*

Table S1. Dichotomized location of aneurysm in anterior and posterior circulation.

Anterior circulation (main vessels and their branches)

- Internal carotid artery (ICA)
- Middle cerebral artery (MCA)
- Anterior cerebral artery (ACA)
- Anterior communicating artery (ACom)
- Carotid branches (ophthalmic artery and anterior choroidal artery)

Posterior circulation (main vessels and their branches)

- Posterior communicating artery (PCom)
- Posterior cerebral artery (PCA)
- Basilar artery
- Superior cerebellar artery (SCA)
- Anterior inferior cerebellar artery (AICA)
- Posterior inferior cerebellar artery (PICA)
- Vertebral artery

According to: Bijlenga P, Ebeling C, Jaegersberg M, Summers P, Rogers A, Waterworth A, Iavindrasana J, Macho J, Pereira VM, Bukovics P, et al. Risk of rupture of small anterior communicating artery aneurysms is similar to posterior circulation aneurysms. Stroke. 2013;44:3018-3026. doi: 10.1161/STROKEAHA.113.001667

Figure S2. Flow diagram of included patients.

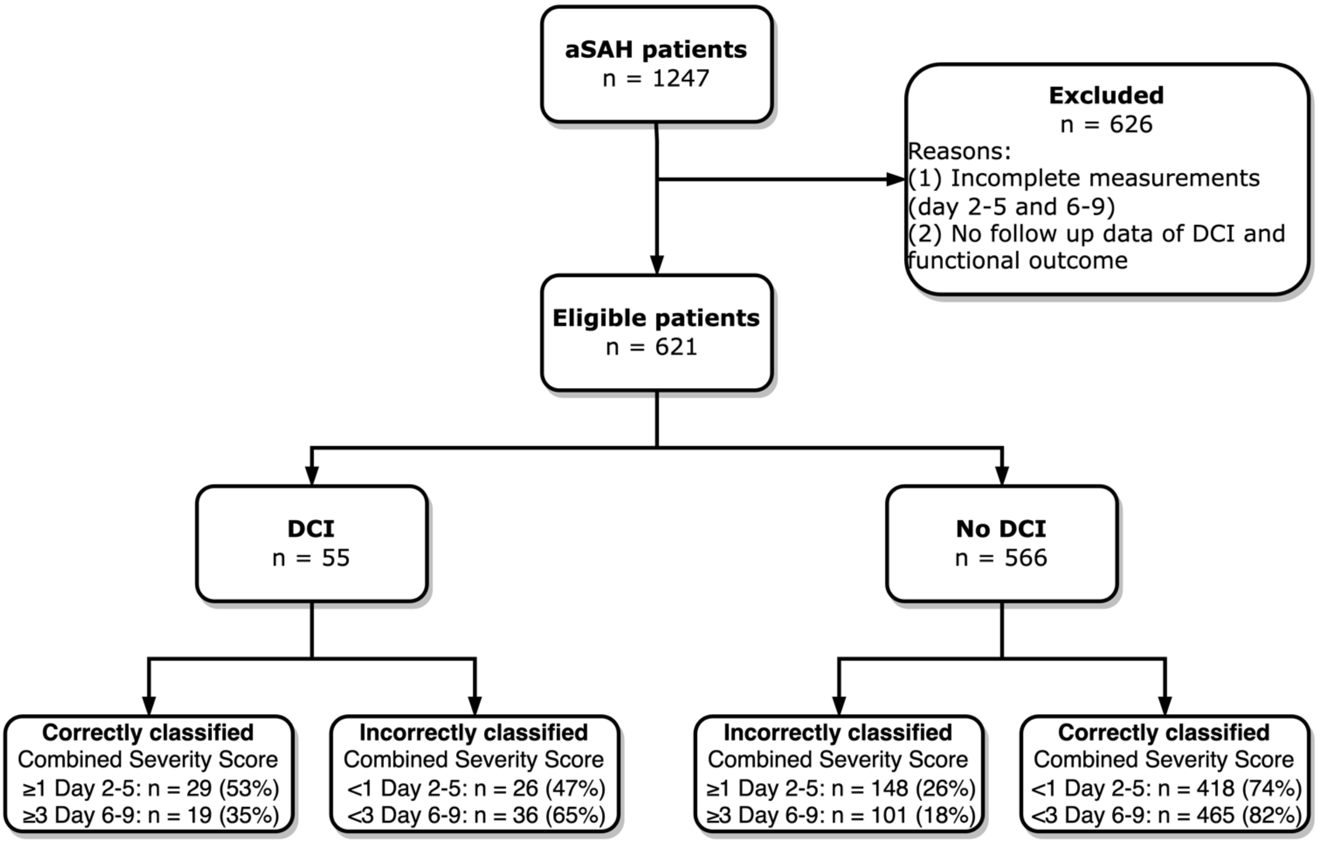

Figure S3. Flow diagram of included patients.
aSAH indicates aneurysmal subarachnoid hemorrhage; DCI, delayed cerebral ischemia.

Table S3. Functional Outcome and Delayed Cerebral Ischemia for included and excluded patients.

	**Included N = 621**	**Exluded N = 626**	**p-value**
Functional Outcome (Binomial modified Rankin Scale)			<0.001
mRS ≤ 3	512 (82%)	434 (69%)	
mRS > 3	109 (18%)	192 (31%)	
Delayed Cerebral Ischemia			0.2
no DCI	566 (91%)	558 (89%)	
DCI	55 (8.9%)	68 (11%)	

Pearson's Chi-squared test, <0.001
DCI indicates Delayed Cerebral Ischemia.

Table S4. Patient Characteristics for DCI and functional outcome.

	Overall N = 621[1]	DCI N = 55[1]	no DCI N = 566[1]	p-value[2]	mRS > 3, N = 109[1]	mRS ≤ 3, N = 512[1]	p-value[2]
Female	428 (69%)	35 (64%)	393 (69%)	0.38	72 (66%)	356 (70%)	0.48
Age, years, median (IQR)	54 (45, 62)	53 (44, 62)	54 (46, 62)	0.65	60 (50, 69)	52 (45, 60)	<0.001
Hypertension	147 (24%)	14 (25%)	133 (23%)	0.74	28 (26%)	119 (23%)	0.59
**World Federation of Neurosurgical Societies grading after neurological resuscitation**				**0.027**			**<0.001**
I	273 (44%)	20 (36%)	253 (45%)		23 (21%)	250 (49%)	
II	183 (29%)	13 (24%)	170 (30%)		26 (24%)	157 (31%)	
III	24 (3.9%)	2 (3.6%)	22 (3.9%)		3 (2.8%)	21 (4.1%)	
IV	104 (17%)	11 (20%)	93 (16%)		38 (35%)	66 (13%)	
V	37 (6.0%)	9 (16%)	28 (4.9%)		19 (17%)	18 (3.5%)	
**Modified Fisher grading scale***				0.18			**<0.001**
1	146 (25%)	7 (14%)	139 (26%)		6 (6.7%)	132 (29%)	
2	133 (23%)	9 (18%)	124 (23%)		11 (11%)	122 (25%)	
3	103 (17%)	10 (20%)	93 (17%)		19 (18%)	84 (17%)	
4	207 (35%)	25 (49%)	182 (34%)		67 (64%)	140 (29%)	
**Location aneurysm***				0.93			0.42
Anterior	416 (67%)	38 (69%)	378 (67%)		68 (62%)	348 (68%)	
Posterior	200 (32%)	17 (31%)	183 (32%)		40 (37%)	160 (31%)	
**Size aneurysm, mm, median (IQR)**	6.0 (4.0, 9.0)	6.0 (4.0, 9.2)	6.0 (4.0, 9.0)	>0.99	6.5 (5.0, 10)	6.0 (4.0, 8.0)	0.13
**Treatment** *				0.46			0.73
Coiling	343 (58%)	27 (53%)	316 (59%)		49 (56%)	294 (58%)	
Clipping	247 (42%)	24 (47%)	223 (41%)		38 (44%)	209 (42%)	
**Drain**				**0.043**			**<0.001**
No drain	188 (34%)	12 (22%)	176 (31%)		13 (12%)	175 (34%)	
Drain before treatment	252 (41%)	19 (35%)	233 (41%)		42 (39%)	210 (41%)	
Drain after treatment	181 (29%)	24 (44%)	157 (28%)		54 (50%)	127 (25%)	
**SAFIRE grade**				**0.049**			**<0.001**
1	229 (37%)	17 (31%)	212 (37%)		12 (11%)	217 (42%)	
2	174 (28%)	10 (18%)	164 (29%)		16 (15%)	158 (31%)	
3	113 (18%)	14 (25%)	99 (17%)		33 (30%)	80 (16%)	
4	98 (16%)	12 (22%)	86 (15%)		44 (40%)	54 (11%)	
5	7 (1.1%)	2 (3.6%)	5 (0.9%)		4 (3.7%)	3 (0.6%)	
**Delayed Cerebral Ischemia**							**<0.001**
DCI	55 (8.9%)				27 (25%)	28 (5.5%)	
No DCI	566 (91%)				82 (75%)	484 (95%)	
**Functional Outcome (Binomial modified Rankin Scale)**				**<0.001**			-
mRS > 3	109 (18%)	27 (49%)	82 (14%)		-	-	
mRS ≤ 3	512 (82%)	28 (51%)	484 (86%)		-	-	

P < 0.05 was considered significant (indicated bold). Where applicable, medians with interquartile range (IQR) are presented.
SAFIRE indicates size aneurysm, age, Fisher grade, world federation of neurosurgical societies grading after resuscitation; mRS, modified Rankin Scale; DCI, Delayed Cerebral Ischemia.
* For Modified Fisher scale there were 32 missing values, for location aneurysm 5, for treatment 31.

Table S5. TCD Variables for Delayed Cerebral Ischemia and no Delayed Cerebral Ischemia.

	DCI N = 55	No DCI N = 566	PValue
Middle Cerebral Artery Mean Flow Velocity (cm/s)			
Day 2-5	101 (81, 140)	82 (68, 108)	<0.001
Day 6-9	139 (95, 180)	103 (73, 141)	<0.001
Lindegaard Ratio			
Day 2-5	3.07 (2.43, 4.04)	2.67 (2.11, 3.46)	0.004
Day 6-9	3.58 (3.00, 5.16)	3.11 (2.29, 4.43)	0.002
Anterior Cerebral Artery Mean Flow Velocity (cm/s)			
Day 2-5	83 (63, 111)	76 (60, 94)	0.043
Day 6-9	100 (71, 138)	82 (64, 111)	0.013
Sloan Ratio			
Day 2-5	2.65 (2.10, 3.32)	2.40 (1.90, 3.03)	0.19
Day 6-9	2.75 (2.29, 4.02)	2.58 (1.99, 3.54)	0.053
Posterior Cerebral Artery Mean Flow Velocity (cm/s)			
Day 2-5	45 (38, 58)	44 (36, 52)	0.18
Day 6-9	52 (45, 64)	44 (36, 56)	0.001

DCI indicates Delayed Cerebral Ischemia.
Medians with interquartile range (IQR) are presented.
The Lindegaard ratio is calculated by the mean flow velocity in the middle cerebral artery divided by the mean flow velocity ipsilateral extracranial internal carotid artery, the Sloan ratio is calculated as the mean flow velocity of the anterior cerebral artery divided by ipsilateral mean flow velocity internal carotid artery.
Missing values for Lindegaard ratio 3, for ACA 20, for Sloan ratio 23 and for PCA 26.

Table S6. Combined Severity Score Day 2-5.

	**Delayed Cerebral Ischemia**		Total
	Yes	No	
**≥1**	29 (4.7%)	148 (24%)	177 (29%)
**<1**	26 (4.2%)	418 (67%)	444 (71%)
Total	55 (8.9%)	566 (91%)	621 (100%)

Pearson's Chi-squared test, p<0.001

Table S7. Combined Severity Score Day 6-9.

	**Delayed Cerebral Ischemia**		Total
	Yes	No	
**≥3**	19 (3.1%)	101 (16%)	120 (19%)
**<3**	36 (5.8%)	465 (75%)	501 (81%)
Total	55 (8.9%)	566 (91%)	621 (100%)

Pearson's Chi-squared test, p=0.003
